# Our Reply

**Published:** 1881-07-20

**Authors:** 


### OUR REPLY.

*Dear Doctor*—To yours of the 9th inst., I will reply that I believe in the law of Septinaries as applicable to pregnancy and many other physiological actions.

Under this rule a lunar month of four weeks or twenty-eight days is the interval from the commencement of one menstrual period to another. The average duration of the menstrual flow is seven days, and the normal or natural duration of pregnancy 40 weeks or 280 days. The period of œstruation or heat in the human female, during which, as a rule, she conceives, is also governed by the same law, and continues seven days immediately after the cessation of the menstrual period.

These are all general rules and liable to occasional exceptions from various causes, and it has been observed that the exceptions or modifications which occur are governed by the same septinary law; so that a case is very apt, when going beyond or falling short of the normal period, to do so by 7, 14 or 21 days, but very seldom beyond 7 or 14 days.

Our obstetric tables differ as to the time from which the 280 days should date—some placing it at the first day of menstruation, others at the close of the period. The truth is, it seems to vary as between the two points, but according to our observation it more frequently dates from the commencement of menstruation; especially is this the case with primpara, who indeed, not unfrequently anticipate this time by one or two septinary periods, (7 to 14 days).

The case you mention, according to septinary laws, should have reached her full time on the 23d of May; but the addition of two septinary periods would throw it to the 6th of June, which seems to fit the case precisely.

The claim of certain modern investigators that conception takes place immediately before menstruation, I do not accept, though some experiments have been submitted which seem to favor that theory. The fact that œstruation occurs in the human female immediately after menstruation, is conclusive to my mind that this is the true period of conception. There are, however, rare exceptions in which the period of heat may be hurried up or postponed. These exceptions are due to causes which hasten or retard the development or maturation of the ovum. Dallying with the other sex, lascivious thoughts, or anything that excites sexual desire, may hasten the functional activity of the organs, and lead to rupture of the Graffian vesicle and to its occasional impregnation in advance of the usual period which, as stated, is during the week immediately succeeding the menstrual flow. At this period the vesicle is mature, and is likely to rupture and to be fecundated by the spermatazoa of the male as a result of the very first sexual act after menstruation. It not unfrequently ruptures prematurely and passes off with the catamenial flux. On the other hand, the maturation of the vesicle may be retarded by ill health, mental anxiety or any cause antagonistic to the sexual appetite.

The above is so much of our reply as applies to the matter upon which the inquiry was made. We publish it because it is an important subject, and one which we suppose will be likely to interest our readers. It is possible that there are those who will differ with us, and that our views will be criticised. If so, our columns are open for remarks, and we will be pleased to hear the views of medical brethren upon the subject.

